# Supplementary material for: Molecular physiology of Antarctic diatom natural assemblages and bloom event reveal insights into strategies contributing to their ecological success
Source: mSystems. 2024 Feb 27;9(3):e01306-23. doi: 10.1128/msystems.01306-23 (PMC10949512; doi:10.1128/msystems.01306-23)
Supplement: Supplemental material — Supplemental text, figures, and tables. [file msystems.01306-23-s0001.docx]

**Supplementary Information for**

Molecular physiology of Antarctic diatom natural assemblages reveals multiple strategies contributing to their ecological success

Carly M. Moreno, Margaret Bernish, Meredith G. Meyer, Zuchuan Li, Nicole Waite, Natalie R. Cohen, Oscar Schofield, Adrian Marchetti*

*Corresponding author: Adrian Marchetti

Email: amarchett@unc.edu

**This PDF file includes:**

Supplemental text

Figures S1 to S9

Tables S1 to S3

**Supplemental text**

**S1.** For example, Luria et al. (1) examined 18S rRNA genes from water samples collected at the four corners of the Pal-LTER sampling grid and detected a 2-fold higher species richness in eukaryotic plankton in the northern WAP compared to the southern WAP. In Northern Marguerite Bay, a 15-year time series demonstrated that in years characterized as having high phytoplankton biomass, large pennate diatoms (>20 μm) dominated the 18S rRNA gene community in early spring (December), followed by large centric diatoms in summer (January) (2, 3). Further linking phytoplankton composition to biogeochemistry along the WAP, Lin et al. (4) conducted high-throughput sequencing of the 18S rRNA gene and determined that out of a diverse plankton community of approximately 464 identified operational taxonomic units (OTUs), only 2-3 OTUs could explain a large majority of the spatial variability in carbon export potential (70-90%).

**S2.** Although not significantly correlated in 2018, a long-term study of bacterial production in the WAP found statistically significant relationships among these three parameters (5). Further, while the BP/PP ratio is low in the WAP relative to the global ocean (6), BP can at times be quite high (7); however, the variability in these relationships makes it difficult to predict BP from either PP or Chl *a* from one year to the next. Finally, in 2018,across the regions we observed a strong negative correlation between Chl *a* (and to a lesser extent PP and BP) and salinity, DIC, phosphate and nitrate(+nitrite) concentrations, indicating a drawdown of nutrients by more abundant, actively growing phytoplankton in fresher surface waters. These results are consistent with findings that high phytoplankton biomass, particularly that of diatoms, is responsible for seasonal nutrient depletions (5, 8).

**S3**. Although pathway level groupings of KOs provide information on broad metabolism, the number of KOs that have an associated MO annotation are typically small; in our study only 38% of KOs had a higher-level module annotation (i.e., 6% of all contigs). While still informative, this low level of annotation makes it difficult to draw definitive conclusions from module expression alone. Therefore, we queried the most variable KOs expressed across the WAP (transcripts with the highest log_2_-normalized variance across sites). Expression of the most variable diatom genes shifted along latitudinal and cross-shelf gradients (Supplemental Fig. S5). Coastal stations in the south had higher transcript levels for genes encoding nucleotide and energy production. Specifically, a pyrimidine precursor biosynthesis enzyme was highly abundant (THI5). Pyrimidines are particularly important in dividing cells as building blocks for nucleic acids, but they are equally important for many biochemical processes, including sugar (UDP) metabolism and vitamin B1 synthesis (9). Increased abundance of iron-containing photosynthesis subunits (PsbN and PsaC) as well as cytochrome c oxidase (COX) subunits, suggestive of increased ATP formation through cellular respiration, indicate conditions conducive to growth. In contrast, northern stations had more transcripts for stress related genes such as E3 ubiquitin-protein ligases and proteasome activator protein. Ubiquitination by E3 ligases regulates cell trafficking, DNA repair, and signaling, mediating the degradation of damaged proteins (10) which are processed by the proteasome.

**S4.** Several lines of evidence for Fe limitation in the WAP region exist. First, a cross-shelf gradient of dissolved and particulate iron has been observed, with particularly low concentrations (< 0.1 nmol kg^-1^) widespread in shelf and slope stations, especially in the northern half of the sampling grid (11). Second, severe Fe limitation has been strongly suggested through the measurement of phytoplankton photophysiology, with decreased photosynthetic energy conversion efficiencies and increased decoupled light harvesting chlorophyll-protein antenna complexes in shelf and slope waters, signatures typical of Fe limitation (12, 13). And third, upwelled UCDW waters in offshore and slope areas result in subsurface phytoplankton maxima (14) in contrast to the surface blooms that dominate nearshore waters. Thus, coastal phytoplankton benefit from waters with entrained Fe from sediments, sea ice, or from sources of meteoric water (glacial melt and precipitation) (11). However, until now, molecular evidence of Fe limitation from ‘omic approaches is generally lacking in this region.

References

1. Luria C, Ducklow H, Amaral-Zettler L. 2014. Marine bacterial, archaeal and eukaryotic diversity and community structure on the continental shelf of the western Antarctic Peninsula. Aquat Microb Ecol 73:107–121.

2. Rozema PD, Venables HJ, van de Poll WH, Clarke A, Meredith MP, Buma AGJ. 2017. Interannual variability in phytoplankton biomass and species composition in northern Marguerite Bay (West Antarctic Peninsula) is governed by both winter sea ice cover and summer stratification: Changing phytoplankton at the coastal WAP. Limnol Oceanogr 62:235–252.

3. Bertrand EM, McCrow JP, Moustafa A, Zheng H, McQuaid JB, Delmont TO, Post AF, Sipler RE, Spackeen JL, Xu K, Bronk DA, Hutchins DA, Allen AE. 2015. Phytoplankton–bacterial interactions mediate micronutrient colimitation at the coastal Antarctic sea ice edge. Proc Natl Acad Sci USA 112:9938–9943.

4. Lin Y, Cassar N, Marchetti A, Moreno C, Ducklow H, Li Z. 2017. Specific eukaryotic plankton are good predictors of net community production in the Western Antarctic Peninsula. Sci Rep 7:14845.

5. Ducklow HW, Schofield O, Vernet M, Stammerjohn S, Erickson M. 2012. Multiscale control of bacterial production by phytoplankton dynamics and sea ice along the western Antarctic Peninsula: A regional and decadal investigation. Journal of Marine Systems 98–99:26–39.

6. Bowman JS, Kavanaugh MT, Doney SC, Ducklow HW. 2018. Recurrent seascape units identify key ecological processes along the western Antarctic Peninsula. Global Change Biology 24:3065–3078.

7. Kim H, Kimbrel JA, Vaiana CA, Wollard JR, Mayali X, Buie CR. 2022. Bacterial response to spatial gradients of algal-derived nutrients in a porous microplate. ISME J 16:1036–1045.

8. Brown MS, Munro DR, Feehan CJ, Sweeney C, Ducklow HW, Schofield OM. 2019. Enhanced oceanic CO2 uptake along the rapidly changing West Antarctic Peninsula. Nat Clim Chang 9:678–683.

9. Bertrand E, Allen A. 2012. Influence of vitamin B auxotrophy on nitrogen metabolism in eukaryotic phytoplankton. Frontiers in Microbiology 3.

10. Richburg JH, Myers JL, Bratton SB. 2014. The role of E3 ligases in the ubiquitin-dependent regulation of spermatogenesis. Semin Cell Dev Biol 30:27–35.

11. Annett AL, Fitzsimmons JN, Séguret MJM, Lagerström M, Meredith MP, Schofield O, Sherrell RM. 2017. Controls on dissolved and particulate iron distributions in surface waters of the Western Antarctic Peninsula shelf. Marine Chemistry 196:81–97.

12. Sherman J, Gorbunov MY, Schofield O, Falkowski PG. 2020. Photosynthetic energy conversion efficiency in the West Antarctic Peninsula. Limnology & Oceanography 65:2912–2925.

13. Carvalho F, Fitzsimmons JN, Couto N, Waite N, Gorbunov M, Kohut J, Oliver MJ, Sherrell RM, Schofield O. 2020. Testing the Canyon Hypothesis: Evaluating light and nutrient controls of phytoplankton growth in penguin foraging hotspots along the West Antarctic Peninsula. Limnology and Oceanography 65:455–470.

14. Garibotti IA, Vernet M, Ferrario ME. 2005. Annually recurrent phytoplanktonic assemblages during summer in the seasonal ice zone west of the Antarctic Peninsula (Southern Ocean). Deep Sea Research Part I: Oceanographic Research Papers 52:1823–1841.

15. Steinberg DK, Ruck KE, Gleiber MR, Garzio LM, Cope JS, Bernard KS, Stammerjohn SE, Schofield OME, Quetin LB, Ross RM. 2015. Long-term (1993–2013) changes in macrozooplankton off the Western Antarctic Peninsula. Deep Sea Research Part I: Oceanographic Research Papers 101:54–70.

16. Kozlowski WA, Deutschman D, Garibotti I, Trees C, Vernet M. 2011. An evaluation of the application of CHEMTAX to Antarctic coastal pigment data. Deep Sea Research Part I: Oceanographic Research Papers 58:350–364.

**Fig. S1**. A) Oceanographic parameters on the northern 600, southern 200 and far southern -100 line of the WAP. B) PCA bi-plot of environmental measurements with clustering of stations according to a latitudinal gradient from North to Far South regions.

**Fig. S2.** Relative abundance of the composition of each major subdivision of plankton. Species included were present in at least 1% of all samples.

**Fig. S3**. Phytoplankton community composition along the WAP as determined from HPLC pigment data using CHEMTAX software with initial pigment ratios established from WAP phytoplankton communities (Kozlowski et al. 2011).


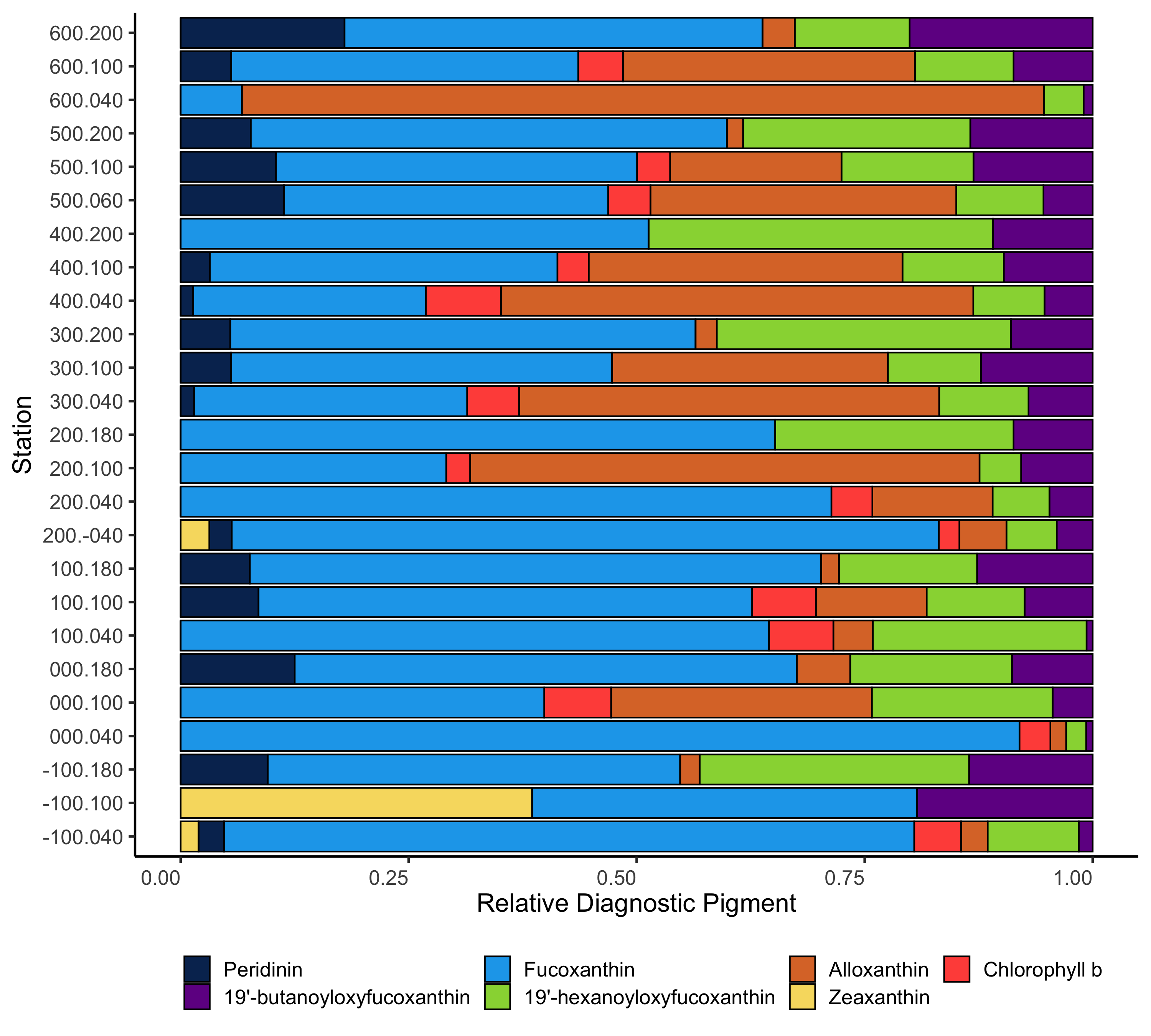

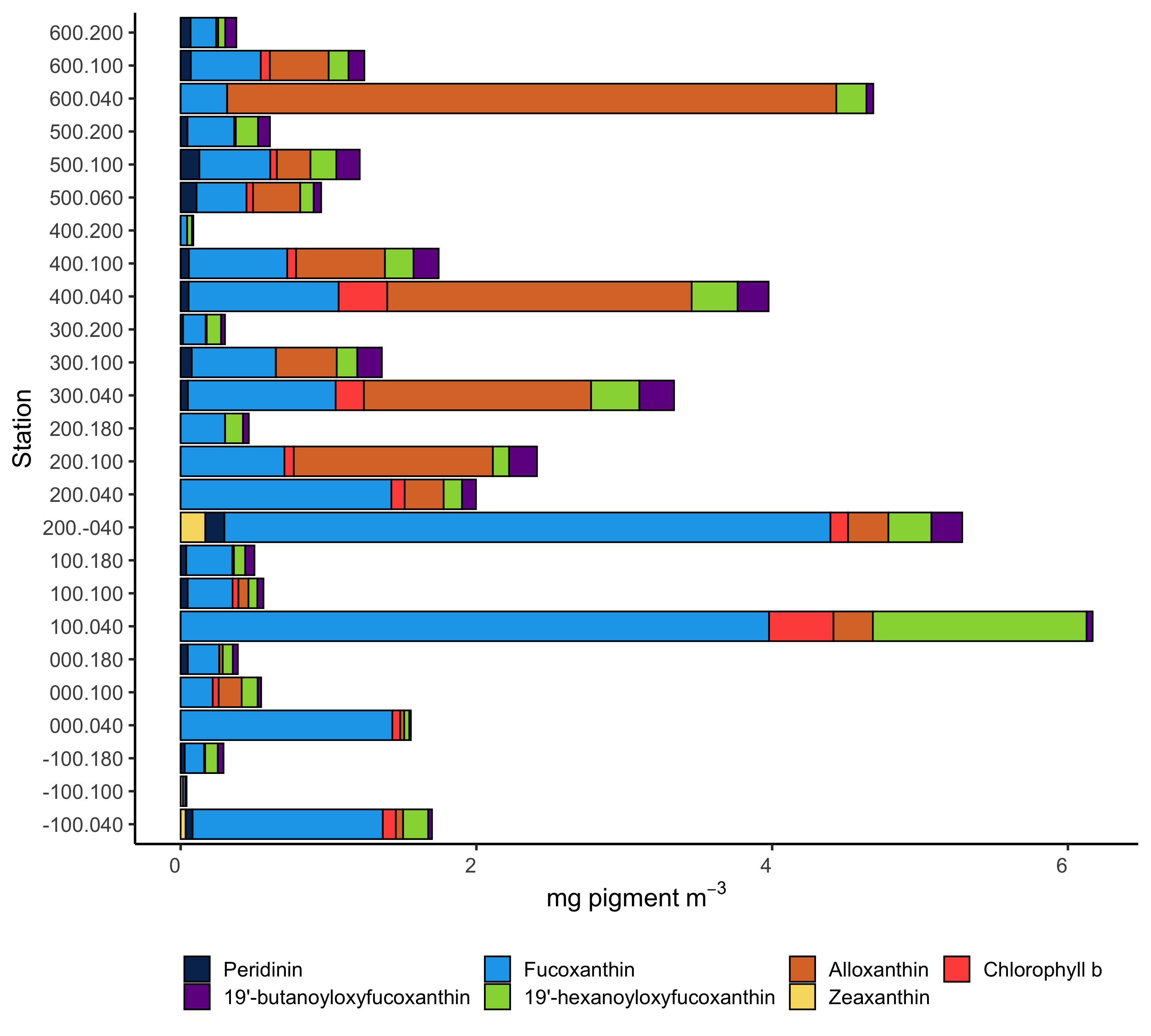

**Fig. S4.** A) Richness and Shannon-Weiner diversity of all eukaryotic phytoplankton across different regions. B) Diatom diversity as a function of different regions along the WAP. Richness not included as equivalent species were found in all regions.

Z-score

**Fig. S5.** Heatmap of log_2_ TPM recruiting to KEGG Class 3 broad scale categories (rows) for all diatom groups, shown as z-scores (TPM deviations from the mean) calculated across samples (columns). Dendrograms show similarity in transcript abundances determined with Euclidean distances and hierarchical clustering. Iron starvation induced proteins (pTF, ISIP1, ISIP2b, ISIP3) manually added as their own module.

log_2_ TPM

**Fig. S6.** Heatmap of log_2_ TPM-normalized diatom transcripts recruiting to the top 50 most variable KO’s (rows) along the WAP. Dendrograms show similarity in transcript abundances determined with Euclidean distances and hierarchical clustering.

log_2_ TPM

**Fig. S7.** Heatmap of log_2_ TPM-normalized *Actinocyclus* sp. transcripts recruiting to the top 50 most variable KO’s (rows). Dendrograms show similarity in transcript abundances determined with Euclidean distances and hierarchical clustering. Stations highlighted in red indicate *Actinocyclus* sp. bloom stations determined from *Actinocyclus* annotations and diatom HPLC fucoxanthin concentrations.

log_2_ TPM

**Fig. S8.** Heatmap of log_2_ TPM-normalized *Actinocyclus* sp. transcripts recruiting to the top 50 most variable KOs (rows) of specific metabolisms; nucleotide and amino acid metabolism, and cofactor and vitamin biosynthesis

**Fig. S9.** Log-transformed TPM of select *Actinocyclus* RHO and THIC genes that indicate active actively growing diatoms. Stations outlined in red indicate bloom locations

**Table S1.** Oceanographic parameters measured during the 2018 Pal-LTER. North-South (NS) regions and East-West (EW) regions are listed for each station according to Steinberg et al. 2015 (15).

| Station | Latitude | Longitude | NS region | EW region | °C | Salinity | DIC (umol kg^-1^) | Nitrite/Nitrate (µmol L^-1^) | Phosphate (µmol L^-1^) | Silicate (µmol L^-1^) | Primary Production (mg C m^-2^ d^-1^) | Chlorophyll *a* (µg L^-1^) | Bacterial Abundance (cell L^-1^) | Bacterial Production (pmol L^-1^ h^-1^) | Phaeopigment (µg L?1) |
| --- | --- | --- | --- | --- | --- | --- | --- | --- | --- | --- | --- | --- | --- | --- | --- |
| 600.040 | -64.93321 | -64.40059 | North | coastal | 2.25 | 33.65 | 2109 | 17.48 | 1.30 | 55.26 | 6.55 | 1.86 | 2.71E+09 | 97.73 | 0.4 |
| 600.100 | -64.57501 | -65.33957 | North | shelf | 0.99 | 33.75 | 2146 | 21.29 | 1.58 | 55.04 | 54.04 | 1.09 | 1.86E+09 | 8.30 | 0.3 |
| 600.200 | -63.9653 | -66.85524 | North | slope | 0.82 | 33.74 | 2157 | 25.42 | 1.68 | 41.96 | 25.55 | 0.41 | 5.19E+08 | 9.59 | 0.11 |
| 500.200 | -64.61099 | -68.29379 | North | slope | 0.52 | 33.74 | 2157 | 24.53 | 1.66 | 38.58 | 8.37 | 0.53 | 3.61E+08 | 7.06 | 0.15 |
| 500.100 | -65.23418 | -66.77544 | North | shelf | 0.87 | 33.70 | 2144 | 21.62 | 1.58 | 55.71 | 14.85 | 1.16 | 1.58E+09 | 33.09 | 0.44 |
| 500.060 | -65.47801 | -66.15043 | North | coastal | 0.98 | 33.59 | 2135 | 19.78 | 1.57 | 57.70 | 29.76 | 1.21 | 1.62E+09 | 5.05 | 0.38 |
| 400.040 | -66.25231 | -67.33584 | North | coastal | 0.03 | 33.19 | 2098 | 18.43 | 1.45 | 57.87 | 30.95 | 4.01 |  | 51.47 | 1.31 |
| 400.100 | -65.87719 | -68.28142 | North | shelf | 0.54 | 33.77 | 2161 | 22.23 | 1.61 | 55.73 | 101.59 | 0.32 | 6.79E+08 | 16.97 | 0.09 |
| 400.200 | -65.23804 | -69.79766 | North | slope | 1.35 | 33.71 | 2150 | 23.58 | 1.57 | 28.43 | 8.63 | 0.28 | 5.29E+08 | 6.83 | 0.09 |
| 300.200 | -65.85005 | -71.37653 | South | slope | 0.93 | 33.72 | 2151 | 24.00 | 1.60 | 28.73 | 8.28 | 0.15 | 2.54E+08 | 9.83 | 0.05 |
| 300.100 | -66.50618 | -69.87708 | South | shelf | 1.03 | 33.74 | 2149 | 23.02 | 1.59 | 44.15 | 23.68 | 0.75 | 5.75E+08 | 12.27 | 0.21 |
| 300.040 | -66.89105 | -68.92263 | South | coastal | 0.51 | 33.26 | 2095 | 16.39 | 1.32 | 57.59 | 48.92 | 1.97 | 6.35E+08 | 0.68 | 0.61 |
| 200.040 | -67.51042 | -70.59103 | South | coastal | 0.43 | 33.32 | 2095 | 14.88 | 1.09 | 38.72 | 78.27 | 2.29 | 7.72E+08 | 10.98 | 0.76 |
| 200.100 | -67.11405 | -71.53085 | South | shelf | 0.71 | 33.60 | 2139 | 19.98 | 1.50 | 52.09 | 64.60 | 2.01 | 7.62E+08 | 5.52 | 0.64 |
| 200.180 | -66.57926 | -72.73886 | South | slope | 1.09 | 33.72 | 2152 | 23.98 | 1.58 | 30.52 | 97.96 | 0.50 | 6.33E+08 | 12.05 | 0.15 |
| 100.180 | -67.15631 | -74.4745 | Far South | slope | 1.16 | 33.77 | 2157 | 24.71 | 1.65 | 36.76 | 19.40 | 0.55 | 2.78E+08 |  | 0.17 |
| 100.100 | -67.70761 | -73.27071 | Far South | shelf | 0.22 | 33.37 | 2128 | 21.83 | 1.54 | 38.25 | 18.63 | 0.42 | 4.14E+08 | 15.50 | 0.15 |
| 100.040 | -68.09312 | -72.38819 | Far South | coastal | 0.22 | 33.12 | 2030 | 5.71 | 0.43 | 39.16 | 162.87 | 6.49 | 1.14E+09 | 74.56 | 2.44 |
| 000.040 | -68.69465 | -74.19341 | Far South | coastal | -1.68 | 33.36 | 2159 | 24.46 | 1.76 | 58.48 | 70.57 | 1.94 | 1.03E+09 | 15.56 | 0.92 |
| 000.100 | -68.27142 | -75.11729 | Far South | shelf | -0.17 | 33.01 | 2121 | 21.51 | 1.51 | 39.00 | 29.35 | 0.57 | 3.95E+08 | 4.22 | 0.24 |
| 000.180 | -67.71603 | -76.29576 | Far South | slope | 0.79 | 33.49 | 2155 | 24.39 | 1.66 | 40.26 | 19.71 | 0.44 | 3.42E+08 | 3.70 | 0.12 |
| -100.180 | -68.25703 | -78.19839 | Far South | slope | 0.63 | 33.62 | 2163 | 24.99 | 1.69 | 36.06 | 14.08 | 0.32 | 3.77E+08 | 4.46 | 0.1 |
| -100.100 | -68.82457 | -77.05133 | Far South | shelf | -0.02 | 33.41 | 2165 | 26.98 | 1.78 | 45.74 | 10.35 | 0.39 | 5.22E+08 | 3.44 | 0.1 |
| -100.040 | -69.25031 | -76.14377 | Far South | coastal | -1.77 | 33.05 | 2117 | 20.88 | 1.45 | 48.24 | 64.72 | 2.16 | 4.45E+08 | 18.54 | 0.79 |
| 200.-040 | -68.03066 | -69.28707 | South | coastal | 1.61 | 33.29 | 2066 | 8.54 | 0.60 | 38.41 | 94.12 | 3.45 | 6.55E+08 | 67.50 | 1.23 |

**Table S2.** 2018 HPLC pigment data using CHEMTAX software with initial pigment ratios established from WAP phytoplankton communities (Kozlowski et al. 2011) (16).

| Station | Peridinin | 19'-butanoyloxyfucoxanthin | Fucoxanthin | 19'-hexanoyloxyfucoxanthin | Alloxanthin | Zeaxanthin | Chlorophyll b |
| --- | --- | --- | --- | --- | --- | --- | --- |
| 600.040 | 0 | 0.047 | 0.314 | 0.203 | 4.12 | 0 | 0 |
| 600.100 | 0.069 | 0.108 | 0.473 | 0.134 | 0.398 | 0 | 0.061 |
| 600.200 | 0.068 | 0.076 | 0.173 | 0.047 | 0.013 | 0 | 0 |
| 500.200 | 0.046 | 0.081 | 0.315 | 0.15 | 0.011 | 0 | 0 |
| 500.100 | 0.127 | 0.158 | 0.479 | 0.175 | 0.228 | 0 | 0.044 |
| 500.060 | 0.108 | 0.051 | 0.338 | 0.091 | 0.319 | 0 | 0.044 |
| 400.040 | 0.054 | 0.21 | 1.014 | 0.31 | 2.059 | 0 | 0.328 |
| 400.100 | 0.056 | 0.17 | 0.665 | 0.194 | 0.6 | 0 | 0.06 |
| 400.200 | 0 | 0.009 | 0.044 | 0.032 | 0 | 0 | 0 |
| 300.200 | 0.016 | 0.027 | 0.153 | 0.097 | 0.007 | 0 | 0 |
| 300.100 | 0.075 | 0.167 | 0.569 | 0.139 | 0.411 | 0 | 0 |
| 300.040 | 0.049 | 0.235 | 0.999 | 0.327 | 1.537 | 0 | 0.19 |
| 200.040 | 0 | 0.095 | 1.425 | 0.124 | 0.263 | 0 | 0.09 |
| 200.100 | 0 | 0.189 | 0.702 | 0.11 | 1.346 | 0 | 0.063 |
| 200.180 | 0 | 0.04 | 0.301 | 0.12 | 0 | 0 | 0 |
| 100.180 | 0.038 | 0.063 | 0.313 | 0.076 | 0.01 | 0 | 0 |
| 100.100 | 0.048 | 0.042 | 0.303 | 0.06 | 0.068 | 0 | 0.039 |
| 100.040 | 0 | 0.041 | 3.979 | 1.445 | 0.267 | 0 | 0.435 |
| 000.040 | 0 | 0.011 | 1.432 | 0.034 | 0.027 | 0 | 0.053 |
| 000.100 | 0 | 0.024 | 0.217 | 0.108 | 0.156 | 0 | 0.04 |
| 000.180 | 0.048 | 0.034 | 0.213 | 0.069 | 0.023 | 0 | 0 |
| -100.180 | 0.028 | 0.039 | 0.131 | 0.086 | 0.006 | 0 | 0 |
| -100.100 | 0 | 0.008 | 0.017 | 0 | 0 | 0.015 | 0 |
| -100.040 | 0.048 | 0.026 | 1.286 | 0.17 | 0.049 | 0.033 | 0.087 |
| 200.-080 | 0.13 | 0.209 | 4.098 | 0.291 | 0.273 | 0.166 | 0.119 |

**Table S3.** Sequencing, assembly, and mapping statistics for metatranscriptomes of each sample.

| Station | Raw sequence reads (million) | Trimmed sequence reads (million) | Number of contigs | Avgerage length (bp) | Maximum length (bp) | Minimum length (bp) | N50 | Mapping efficiency to combined assembly (%) |
| --- | --- | --- | --- | --- | --- | --- | --- | --- |
| 600.040 | 16.3 | 15.9 | 615,687 | 410 | 9704 | 73 | 414 | 81.6 |
| 600.100 | 14.6 | 14.2 | 774,947 | 409 | 7386 | 78 | 400 | 74.9 |
| 600.200 | 14.8 | 14.3 | 729,388 | 386 | 5609 | 73 | 365 | 71.8 |
| 500.200 | 14.2 | 13.8 | 698,566 | 389 | 7552 | 76 | 364 | 70.4 |
| 500.100 | 14.5 | 14.1 | 737,581 | 410 | 6198 | 74 | 394 | 72.1 |
| 500.060 | 13.7 | 13.4 | 592,842 | 425 | 11939 | 75 | 429 | 80.6 |
| 400.040 | 14.2 | 13.8 | 655,372 | 421 | 12191 | 74 | 412 | 73.8 |
| 400.100 | 13.4 | 13.1 | 709,953 | 383 | 6310 | 78 | 357 | 78.0 |
| 400.200 | 14.1 | 13.7 | 632,835 | 379 | 5551 | 74 | 359 | 69.3 |
| 300.200 | 15.0 | 14.6 | 796,624 | 411 | 8601 | 75 | 399 | 73.2 |
| 300.100 | 16.8 | 16.4 | 675,170 | 389 | 6179 | 75 | 376 | 75.8 |
| 300.040 | 14.9 | 14.5 | 714,397 | 373 | 5293 | 73 | 345 | 77.8 |
| 200.040 | 14.6 | 14.2 | 644,421 | 399 | 7363 | 75 | 381 | 70.3 |
| 200.100 | 12.9 | 12.5 | 626,118 | 412 | 6220 | 76 | 404 | 73.3 |
| 200.180 | 14.2 | 13.8 | 648,686 | 402 | 5875 | 73 | 388 | 74.3 |
| 100.180 | 15.8 | 15.4 | 350,342 | 404 | 6473 | 74 | 386 | 78.2 |
| 100.100 | 13.8 | 13.5 | 486,601 | 376 | 4778 | 73 | 359 | 77.0 |
| 100.040 | 13.3 | 12.9 | 660,588 | 405 | 5460 | 73 | 397 | 80.9 |
| 000.040 | 13.2 | 12.9 | 745,478 | 425 | 6544 | 76 | 424 | 72.3 |
| 000.100 | 14.4 | 14.0 | 695,824 | 406 | 7490 | 73 | 388 | 78.2 |
| 000.180 | 101.8 | 99.6 | 792,433 | 406 | 7697 | 73 | 395 | 75.3 |
| -100.180 | 14.4 | 14.0 | 607,556 | 389 | 7008 | 74 | 375 | 73.8 |
| -100.100 | 17.1 | 16.6 | 711,812 | 386 | 7540 | 76 | 362 | 76.1 |
| -100.040 | 14.3 | 13.9 | 350,342 | 373 | 4778 | 73 | 345 | 76.8 |
| 200.-040 | 16.2 | 15.7 | 796,624 | 425 | 12191 | 78 | 429 | 71.5 |
